# Cognitive Behavioral Therapy for Insomnia as Adjunctive Therapy to Antipsychotics in Schizophrenia: A Case Report

**DOI:** 10.3389/fpsyt.2018.00260

**Published:** 2018-06-12

**Authors:** Muneto Izuhara, Hiroyuki Matsuda, Ami Saito, Maiko Hayashida, Syoko Miura, Arata Oh-Nishi, Ilhamuddin Abdul Azis, Rostia Arianna Abdullah, Keiko Tsuchie, Tomoko Araki, Arauchi Ryousuke, Misako Kanayama, Sadayuki Hashioka, Rei Wake, Tsuyoshi Miyaoka, Jun Horiguchi

**Affiliations:** Department of Psychiatry, Faculty of Medicine, Shimane University, Izumo, Japan

**Keywords:** schizophrenia, insomnia, cognitive behavioral therapy, long acting injectable antipsychotic(LAI), cognitive behavioral therapy for insomnia(CBT-i)

## Abstract

The authors present the case of a 38-year-old man with schizophrenia and with severe insomnia, who attempted suicide twice during oral drug therapy with risperidone. The patient slept barely 2 or 3 h per night, and he frequently took half days off from work due to excessive daytime sleepiness. As a maladaptive behavior to insomnia, he progressively spent more time lying in bed without sleeping, and he repeatedly thought about his memories, which were reconstructed from his hallucinations. His relatives and friends frequently noticed that his memories were not correct. Consequently, the patient did not trust his memory, and he began to think that the hallucinations controlled his life. During his insomniac state, he did not take antipsychotic drugs regularly because of his irregular meal schedule due to his excessive daytime sleepiness. The authors started cognitive behavioral therapy for insomnia (CBT-i) with aripiprazole long acting injection (LAI). CBT-i is needed to be tailored to the patient's specific problems, as this case showed that the patient maladaptively use chlorpromazine as a painkiller, and he exercised in the middle of the night because he believed he can fall asleep soon after the exercise. During his CBT-i course, he learned how to evaluate and control his sleep. The patient, who originally wanted to be short sleeper, began to understand that adequate amounts of sleep would contribute to his quality of life. He finally stopped taking chlorpromazine and benzodiazepine as sleeping drugs while taking suvorexant 20 mg. Through CBT-i, he came to understand that poor sleep worsened his hallucinations, and consequently made his life miserable. He understood that good sleep eased his hallucinations, ameliorated his daytime sleepiness and improved his concentration during working hours. Thus, he was able to improve his self-esteem and self-efficacy by controlling his sleep. In this case report, the authors suggest that CBT-i can be an effective therapy for schizophrenia patients with insomnia to the same extent of other psychiatric and non-psychiatric patients.

## Background

Sleep disturbance occurs in up to 80% of patients with schizophrenia (1–3) in the prodromal phase or precedes psychotic exacerbation (4). Patients with schizophrenia have various sleep abnormalities such as increased sleep onset, decreased total sleep time, more waking time after sleep onset, decreased sleep efficacy, reduced slow wave sleep, reductions in latency and duration of rapid eye movement (REM) sleep (5, 6). Furthermore, due to circadian rhythm misalignment ranging from phase-advance/delay to non-24 h periods in sleep-wake cycles, highly irregular and fragmented sleep epochs are also common in schizophrenic patients (7, 8). These sleep and circadian disturbances are also observed early or in the prodromal stages of psychosis (9, 10). Poor sleep and a disturbed circadian rhythm cause cognitive dysfunction, low treatment adherence, poor family relationships, and poor self-efficacy, and there is an increasing awareness of the importance of sleep to mental health (11–13). Cognitive behavioral therapy for insomnia (CBT-i) is a promising technique to improve not only sleep abnormalities but also a patient's self-esteem because patients have to think about and discover their own problems during the CBT-i course (14). This procedure makes patients feel like they are participating in their own therapy, compared to just answering questions asked by and taking medications prescribed by physicians (15). Nevertheless, monotherapy of sleep medication or CBT-i fails to improve patients' psychotic symptoms (16–18). Meanwhile, long-acting injection (LAI) of antipsychotic drugs is available and these drugs have produced improvements in psychiatric symptoms and reduced relapses and the rate of hospitalization (19–23). In this report, the authors present a case in which CBT-i was effective in the treatment of a schizophrenic patient with severe insomnia who had twice attempted suicide. The authors believe that this case suggests that schizophrenia patients can benefit from CBT-i, as other psychiatric and non-psychiatric patients do.

## Case presentation

**Figure 1:**
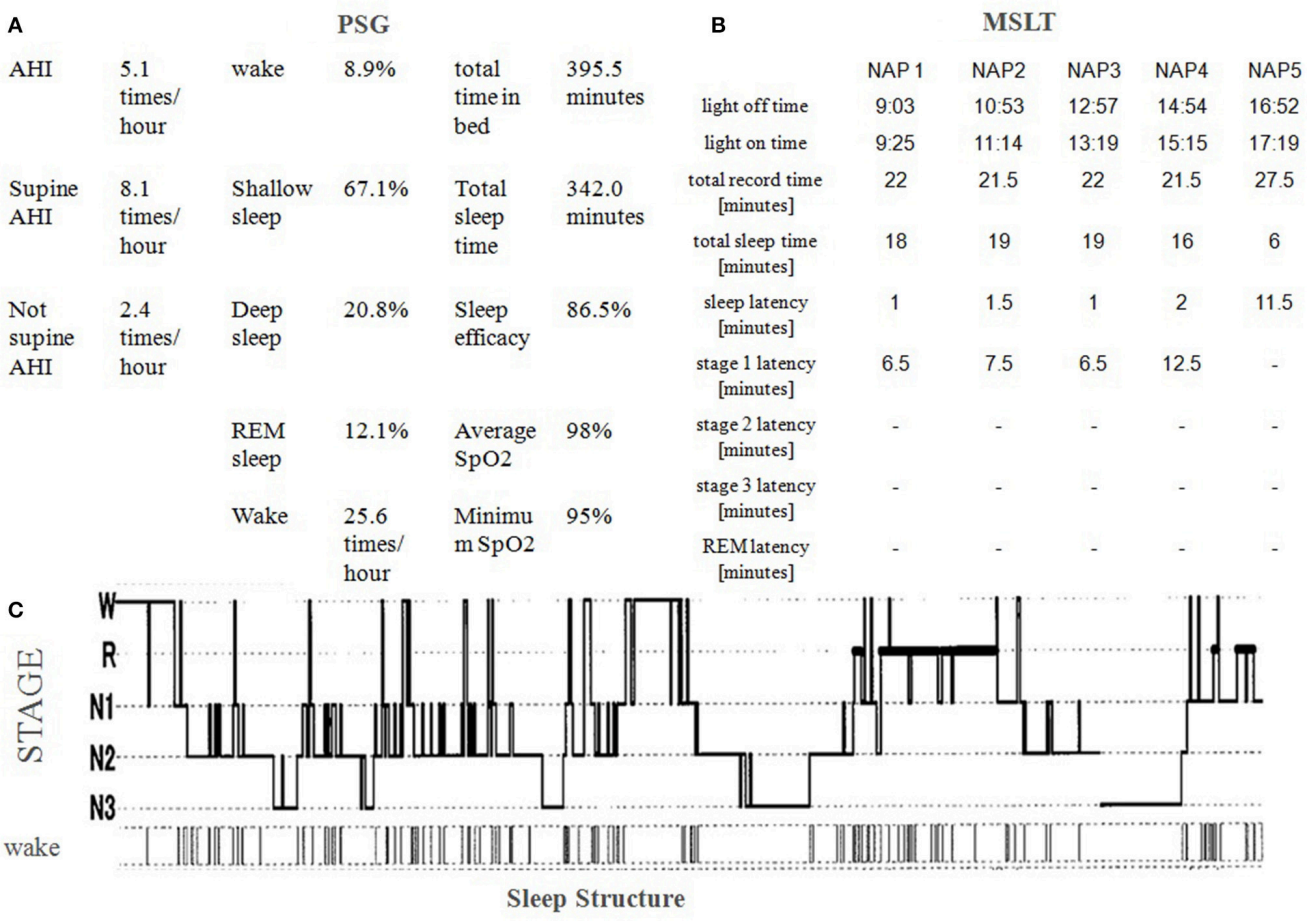
**(A)** Polysomnography (PSG) results show slightly elevated Apnea-Hypopnea Index (AHI; 5.1 times/hour) and severe night awakening (25.6 times/hour). Minimum SpO2 (95%) was not severely decreased. Stage 1 Non REM sleep was slightly increased and REM sleep slightly decreased. Stage 3 sleep and sleep efficacy were normal. **(B)** A multiple sleep latency test (MSLT) shows excessive daytime sleepiness. He slept within 2 min four times out of five. Narcolepsy was excluded because no sleep onset REM (SoREM) was observed. **(C)** Sleep structure shows he woke a lot at the beginning of his sleep and woke an astonishing number of times. He took chlorpromazine 12.5 mg three times at 22:10, 0:15, and 2:45. No snoring of no periodic limb movement was observed.

**Figure 2:**
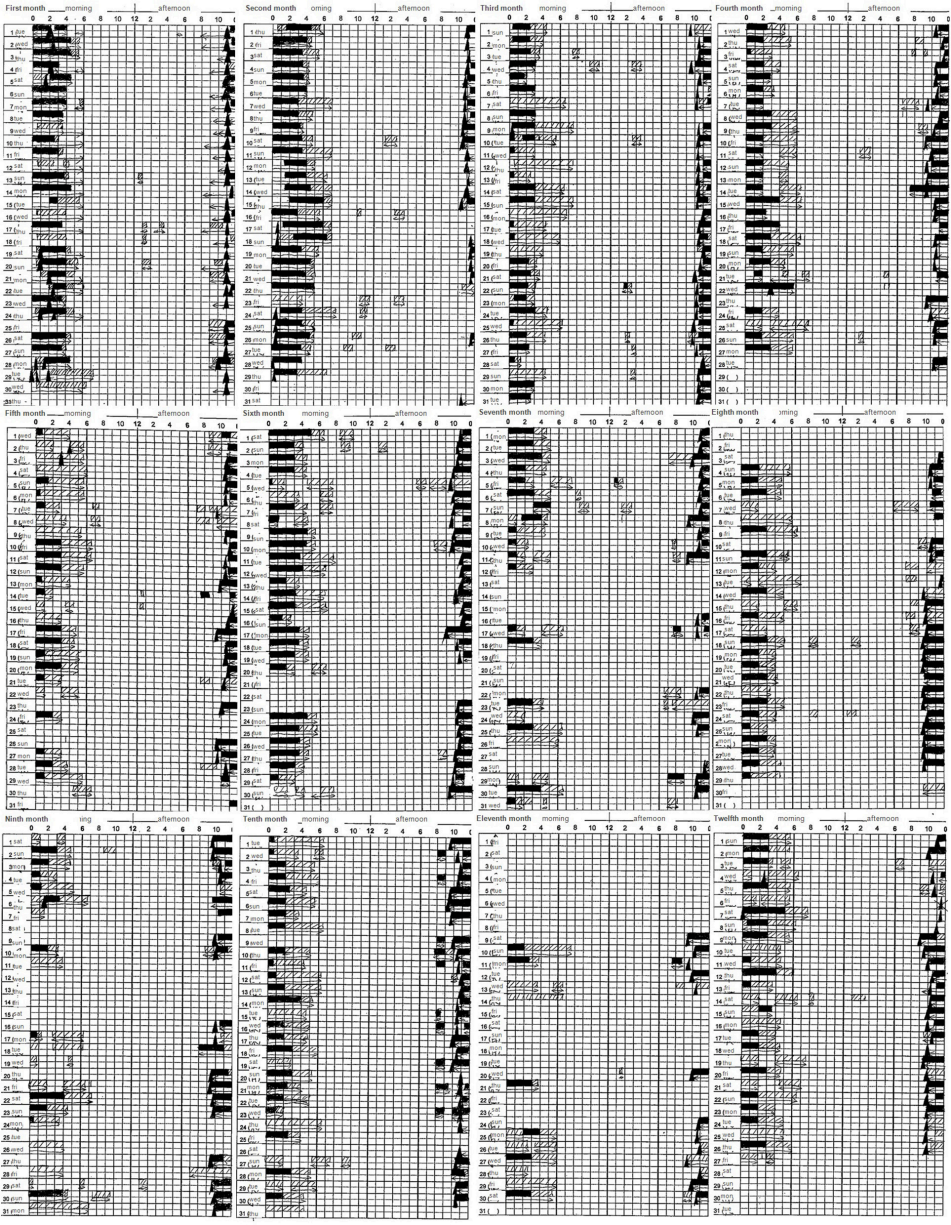
Sleep log: ■ represents sleep, □ represents drowsiness, and ▲ represents taking a sleeping pill. Sleep time, including drowsiness, steadily increased from 5 to 7 h. No sleep phase advance/delay was observed.

**Figure 3:**
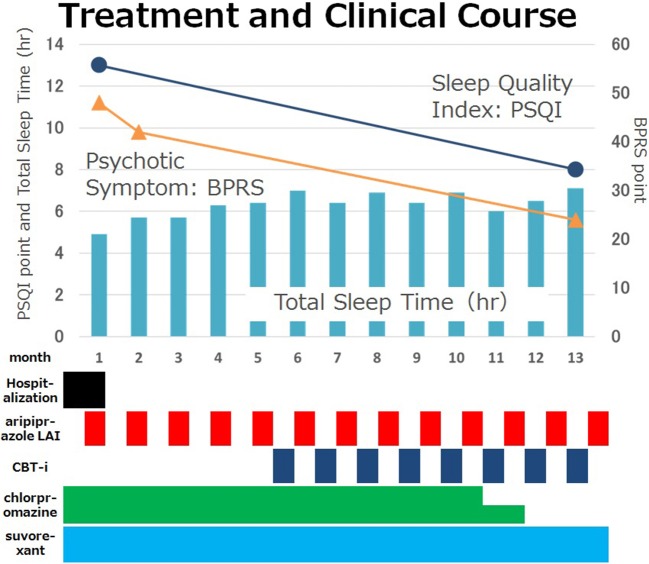
Case treatment and clinical course. The patient's total sleep time increased from 5 to 7 h per day and his insomnia and psychotic symptoms decreased steadily. He could stop using chlorpromazine as a sedative. Psychiatric symptoms were evaluated by BPRS and sleep disturbance was evaluated by PSQI. BPRS dropped from 48 to 24. PSQI dropped from 13 to 8. CBT-i, Cognitive Behavioral Therapy for insomnia; BPRS, The Brief Psychiatric Rating Scale; PSQI, Pittsburgh Sleep Quality Index.

A 38-year-old man with schizophrenia presented after his second suicide attempt through an overdose with 48 tablets of burotizolam, 42 tablets of haloxazolam and 14 tablets of levomepromazine. The patient's childhood and adolescent development was normal. He was a good student and an active soccer player in high school. His social skills were standard, and he had no family history of mental illness. When he was 23 years old and a fourth year university student, he became convinced that he was being observed and he withdrew from social activities. His parents brought him to a psychiatric hospital, and he was diagnosed with schizophrenia according to DSM-IV-TR (24). The prescribed medication worked well and he was able to graduate from university at 27 years old. After graduating, he worked part time in a convenience store or at a nursery for several years. He then started to work at a distribution business under a handicapped employment program. His father committed suicide 3 years before he first presented at our hospital and a friend also died from a sickness. Because his auditory hallucinations repeatedly told him that he was responsible for their deaths, he could not stop blaming himself for their passing, in spite of his mother and brother telling him that he was not responsible. He was pessimistic about his future partly because he was able to earn only a meager income. In order to increase his income, he started a second part-time job at a supermarket in addition to his distribution job. He slept less and felt the accumulation of fatigue. He started to stockpile sleeping medications and he eventually took 76 tablets of brotizolam and 30 tablets of eszopiclone. The next morning his mother found him unconscious and called an ambulance. His mother brought his empty medicine containers to the hospital. At his first presentation, his physical examinations and vital signs were normal. He appeared to be very sleepy, but he managed to speak. The emergency department doctor ordered a blood test, a chest x-ray, an electrocardiogram test, a urine toxicology test, and a computed tomography brain scan. All results were within normal range, except a positive result for benzodiazepine in his urine and a slightly elevated white blood cell count (10.92 × 10^3^/μL). The emergency doctor enlisted a psychiatric doctor to evaluate his mental state. The patient claimed that his auditory hallucinations sounded like someone was booing in addition to radio sounds from a distance. He also claimed he was being tracked by the police. He admitted suicidal ideation and reported that he was sad because he could not die. Because his depressive symptoms occurred 4 weeks prior to his first admission, the authors carefully excluded the possibility of schizoaffective disorder and depressive disorders or bipolar disorder. However, the patient did not show manic symptom or markedly diminished interest, and his depressive thoughts seemed to ease shortly after his admission. Obviously, his mood episodes have been present for a minority of the total duration of the active and residual phases of illness; however, his memory changing delusion and auditory hallucination remains continuously. Furthermore, he showed negative symptom that he had withdrew from social activity except working. The authors diagnosed schizophrenia according to DSM 5 (25). His decreased ability to discriminate between his thought and true memories as mentioned previously suggests the presence of disturbance of the self which also supports this diagnosis (26). The authors prescribed risperidone 6 mg, brotizolam 0.25 mg, and eszopiclone 2 mg. Soon after the treatment started, he became calm and claimed his suicidal ideation disappeared. However, during the patient's second hospitalization, 6 months later, he admitted that he had lied. He wanted to go home quickly so he pretended to be healthy. He subsequently obtained a distribution job contract for the coming season by himself and he was supposed to be followed by a nearby clinic as a condition of his hospital discharge. He started his distribution job but he could not work regularly. Again, he wanted to earn more money so he started attending lectures to get a healthcare worker license. Consequently, his sleep time was reduced and he started to feel life was troublesome once again. He subsequently overdosed as mentioned previously. The next morning, his mother brought him to the emergency department again. She had no idea when he attempted to commit suicide but she last saw him the previous night at 10 p.m. His mother brought his empty medicine containers. His vital signs were normal, and he managed to speak. The emergency doctor conducted a blood test, a chest x-ray and a computed tomography brain scan. All the results were normal, except an elevated white blood cell count (12.16 × 10^3^/μL), creatine kinase (429 U/L), and chloride (109 mmol/L). His mother brought with her more than 100 risperidone tablets. It became obvious that he had not taken his pills regularly. The authors thought his adherence worsened during his psychotic period and started a long acting injectable antipsychotic (LAI). Because the patient worked regularly, the authors choose an injection given once in a 4-week period. Furthermore, because several studies showed it made significant improvements in the quality of life (22), the authors chose aripiprazole LAI at 400 mg. The authors also prescribed 20 mg of suvorexant per day and gradually discontinued brotizolam 0.25 mg and flunitrazepam 2 mg because the authors were concerned about a possible third suicide attempt while using benzodiazepine. Because both of the patient's admissions were associated with poor sleep, the authors examined the patient by polysomnography (PSG) and a multiple sleep latency test (MSLT) to exclude comorbid diseases such as sleep apnea syndrome or restless legs syndrome. As shown in Figure 1, he woke frequently during his sleep (25.6 times per hour on average as shown in Figure 1A) and he lived with excessive daytime sleepiness (he fell asleep within 2 min; on four out of five trials during the MSLT, as shown in Figure 1B). His Apnea-Hypopnea Index (AHI) was slightly elevated (5.1 times/hour), and respiratory events were not associated with significant desaturations (the minimum SpO2 was 95%). His BMI was 19.8. Malocclusion or tonsil swelling was not observed. Figure 2 shows the patient's sleep log. The patient did not show sleep phase advance or delay. The patient's Pittsburgh Sleep Quality Index (PSQI) score was 13, while over 5 points on the PSQI represents insomnia (27). Two months after his second admission, he was discharged while being prescribed suvorexant 20 mg, and chlorpromazine 25 mg per day in addition to aripiprazole LAI 400 mg per month. His Brief Psychiatric Rating Scale (BPRS) (28) dropped form 48 at admission to 42 at discharge. Six months after his second admission, the authors and the patient started CBT-i according to the CBT-i therapeutic manual (29). The authors also referred to the four causes cited by Chiu et al. (30): (a) beliefs that sleep problems cannot be changed; (b) trauma and adversity; (c) lifestyle choices and lack of motivation; (d) medication side effects and the 12 problems cited by Waite et al. (31): (a) Poor sleep environment; (b) Lack of daytime activity; (c) Lack of evening activity; (d) Disrupted circadian rhythm; (e) Sleep as an escape from distressing experiences; (f) Fear of bed; (g) Nightmares; (h) Night-time awakenings; (i) Sleep disrupted by voices/paranoia; (j) Worry; (k) Neuroleptic medication side effects; and (l) Reducing hypnotics. Our CBT-i consisted of eight sessions with each session ranging from 30 to 45 min. The first two sessions were educational sessions that attempted to find disturbances such as a misunderstanding of sleep hygiene or an inadequate sleep environment. In the other six sessions the authors and the patient tried to find other targets to tackle. For instance, the patient tried eating a carbohydrate (banana) before sleep, stopped checking his watch, warmed his body before going to bed, turned off small lights in his room, changed his routine of taking a bath before eating dinner to prevent him from taking a nap after dinner, bought a blackout curtain and an air conditioner. He also tried to wake up early in order to exercise in the morning instead of doing in the middle of the night because he believed he can fall asleep soon after the exercise. The whole course of sleep and psychological tendencies are shown in Figure 3. The patient's BPRS dropped to 24 and his PSQI dropped to 8. His sleep time increased steadily however, at his sixth session, he claimed that he could not sleep at night and he felt a strong sense of sleepiness during the day. His mental health care team consisted of two physician groups; with one group treating his psychiatric symptoms and the other group (the authors) treating his sleep abnormality. The first physician group increased the patient's chlorpromazine from 25 to 37.5 mg. The authors, as the second physician group treating the patient's sleep abnormality, discussed reducing the patient's chlorpromazine with the first physician group because the authors believed that his sleep troubles were not caused by a difficulty in falling asleep but by the dosage of chlorpromazine being too high for the patient's current ability to fall asleep which was gradually being strengthened by CBT-i. At the seventh session, the authors encountered another misunderstanding of the patient in which the authors believed the patient's headaches were being caused by a lack of sleep, while the patient used chlorpromazine as a painkiller. The authors prescribed acetaminophen 400 mg as a painkiller, and stopped the administration of chlorpromazine. At the eighth session, the patient claimed that he had almost no trouble sleeping except when he forgets to take suvorexant.

## Discussion

As this case showed, schizophrenic patients can benefit from CBT-i. In addition to preventing a recurrence, the patient could stop taking chlorpromazine and benzodiazepine as soporifics while continuing with suvorexant 20 mg. The authors want to emphasize the possibility of using the CBT-i therapy to avoid the polypharmacy caused by severe sleep/circadian disturbance with schizophrenia. Our case with schizophrenia exhibited illogical attitudes toward sleep. CBT-i might be time-consuming, but it is an effective procedure to correct a patient's misunderstandings that might delay recovery. In limitation, the authors cannot exclude the possibility of LAI alone preventing a recurrence, but CBT-i might also strengthen the stability of the prevention from recurrence through enforcing the patient's feelings of self-efficacy by controlling his own life.

Recovery is hard to define (32) because it defines the well-being of each individual patient. LAI treatment improved remission rates from ~42 to 64% and decreased relapse rates from 42 to 9.3% compared with oral antipsychotics (33). However, remission does not mean recovery. It means the patient does not meet diagnostic criteria, but it does not indicate the patient's well-being or recovery. Schizophrenic patients prefer CBT-i therapy to drug therapy because this choice empowers them by allowing them to take responsibility for their own recovery (15).

Insomnia and delusions affected our patient's life. In the state of insomnia, he suffered from delusions repeatedly; therefore, he could not maintain his level of concentration during his work. In his view, sleepiness and hallucinations controlled his life. CBT-i worked very well, partially because LAI eased the patient's hallucinations and delusions and this change made his sleep problems more easily treatable.

During the courses of CBT-i treatment, the authors and the patient focused mainly on the four causes cited by Chiu et al. (30), and the 12 problems cited by Waite et al. (31). For instance, as Chiu's fourth cause, the medication side effects were evident as the source of the true problem of sleep disturbance in the sixth session, and the authors and the patient could therefore agree to stop chlorpromazine. Furthermore, this discontinuation of regular use of chlorpromazine also made the authors noticed that the patient's maladaptive use of antipsychotic drugs as painkiller. Consequently, the patient could stop the use of chlorpromazine completely. Based on Waite's first problem involving a poor sleep environment, the patient bought a new blackout curtain and an air conditioner. Waite's third problem involving a lack of evening activity led us to change the patient's routine around dinner, and thus prevent napping after eating. These kinds of time consuming, persistent efforts improved not only his sleep quality but also his self-esteem.

## Concluding remarks

Like this patient, schizophrenia patients can benefit from CBT-i, as other psychiatric and non-psychiatric patients do. This means that having a psychotic diagnosis is not a criterion for exclusion from CBT-i. This approach, in combination with pharmacological treatment, may offer psychotic patients an additional advantage in terms of sleep quality and overall quality of life, when compared to anti-psychotic drug treatment alone. During the CBT-i course, the patient's sleep stabilized. Furthermore, if CBT-i is properly tailored to the patient, physicians and patients will benefit from finding cognitive distortions or maladaptive behaviors, as this case showed that the authors managed to reduce the use of antipsychotics through correcting the fallacious use of antipsychotic drugs as a painkiller. The authors cannot determine the ratio of the contributions of CBT-i and LAI, but the authors believe that CBT-i was needed to stabilize psychiatric symptoms through strengthening the patient's sleep and self-efficacy. The authors suggest that physicians and patients consider the possibility that schizophrenia with severe sleep disturbance can be ameliorated through this treatment of the sleep disturbance. This case report is a demonstration of the efficacy of the combination of CBT-i and anti-psychotic drugs in schizophrenia with a comorbid insomnia disorder.

## Ethics statement

This case study was carried out in accordance with the recommendations of the Ethical Committee of Shimane University Faculty of Medicine with written informed consent from the subject. The subject gave written informed consent in accordance with the Declaration of Helsinki. Written informed consent was also obtained from the patient for the publication of this case report.

## Author contributions

MI, HM, MH, SM, TM: substantial contributions to the conception or design of the work; MI, AS, IA, RA, KT, TA, AO-N, RAA, and MK: acquisition, analysis, or interpretation of data for the work; MI, HM, SH, RW, TM, and JH: drafting the work or revising it critically for important intellectual content; MI, HM, AS, MH, SM, AO-N, IA, RA, KT, TA, RAA, MK, SH, RW, TM, and JH: final approval of the version to be published.

### Conflict of interest statement

The authors declare that the research was conducted in the absence of any commercial or financial relationships that could be construed as a potential conflict of interest.
